# Counteracting Influenza Virus Evolution Through the Full Spectrum of Broadly Protective Antibodies

**DOI:** 10.1111/imr.70147

**Published:** 2026-07-29

**Authors:** Yu Adachi, Yoshimasa Takahashi

**Affiliations:** ^1^ Research Center for Vaccine Development, National Institute of Infectious Diseases, Japan Institute for Health Security Tokyo Japan

**Keywords:** broadly protective antibody, mechanism of action, next‐generation influenza vaccines

## Abstract

Despite the presence of licensed vaccines and therapeutics, influenza viruses remain a major public health concern, as they cause seasonal respiratory infections and pose a pandemic threat through the emergence of zoonotic strains. Although neutralizing antibodies elicited by seasonal vaccination constitute a primary defense against infection, their protective capacity is often limited because they frequently target the epitopes that are highly susceptible to structural changes through viral evolution. To overcome this challenge, broadly protective antibodies have been intensively investigated in both humans and animal models. These antibodies recognize conserved epitopes and confer protection through multiple mechanisms beyond conventional neutralization. Recent advances in antibody discovery technologies and structural biology have enabled high‐resolution mapping of conserved epitopes and the on‐target antibodies that bind them. Furthermore, although broadly protective antibodies are typically elicited only at low frequencies following standard vaccination, several settings have been reported in which their induction is enhanced. Those findings increase the feasibility of epitope‐focused vaccine strategies for enhancing the breadth and durability of antibody responses. In this review, we summarize current knowledge of broadly protective flu antibodies and discuss how mechanistic and structural insights can guide the development of next‐generation influenza vaccines that offer broad‐spectrum protection against antigenically diverse viruses.

## 1 Introduction

Seasonal influenza remains a major global health burden, causing substantial morbidity and mortality, particularly among vulnerable populations such as children, the elderly, and immunocompromised individuals [1, 2]. In addition to its seasonal epidemics, influenza viruses have caused several pandemics over the past century and outbreaks of highly pathogenic zoonotic strains in the 21st century [3, 4, 5]. To counter the threat of the flu virus, one of the key medical countermeasures is a vaccine that elicits protective immunity and prevents severe disease. Innate immune cells in infected hosts detect viral components through pattern‐recognition receptors, such as toll‐like receptors, and rapidly produce type I and III interferons, along with inflammatory cytokines [6, 7]. These signals establish an antiviral state that restricts viral replication and dissemination, thereby serving as the first line of defense. In contrast, intramuscular vaccines widely used for routine vaccination are not aimed at strengthening the innate immune responses in the local mucosal sites at the nasal cavity and respiratory tract. Rather, they are intended to activate adaptive immunity at systemic sites, which constitute the second line of defense. Adaptive immunity orchestrates humoral immunity mediated by B cells and antibody production, as well as T‐cell‐mediated immunity [8].

A key feature of adaptive immunity is the formation of long‐lasting immunological memory during the initial encounter with viruses. Memory B cells and T cells mediate robust and rapid recall responses upon re‐encounter by homologous virus strains. Furthermore, humoral immunity can increase its binding affinity for pathogens through somatic hypermutation of immunoglobulin genes and clonal selection, thereby improving the protective function per molecule [9, 10]. Indeed, the concentration of influenza antibodies, specifically those that neutralize viruses or inhibit hemagglutination by the viral envelope hemagglutinin (HA) protein, correlates relatively well with protection [11, 12]. Therefore, the primary aim of current influenza vaccination is to induce neutralizing and hemagglutination inhibition (HAI) antibodies, widely used immune correlates of protection (CoP).

Influenza infection and vaccination elicit antibody responses against various viral antigens, including envelope and internal proteins [13]. Of those viral antigens, the HA envelope glycoprotein is the primary target of neutralizing antibodies. Antibodies elicited by routine vaccination primarily bind to the HA head domain at the membrane‐distal site that engages the binding to the cellular receptor, sialic acid. Many HA head antibodies have the potential to block viral attachment and entry by inhibiting host receptor‐binding and include those with potent neutralizing and HAI activity [14, 15]. However, the HA head epitopes targeted by such neutralizing (HAI^+^) antibodies are hypervariable and tolerate antigenic plasticity, thereby enabling viruses to escape through mutations. This virus strategy is the roadblock that needs to be overcome by vaccine‐elicited protective antibodies. A breakthrough discovery was made in the early 1990s by Okuno et al., which isolated murine monoclonal antibodies with broadly neutralizing activity against Group 1 influenza A strains [16, 17]. Subsequent structural analyses revealed that these antibodies target a conserved epitope in the HA stalk domain at the membrane‐proximal site [18]. This region is functionally important for viral infection processes and is therefore less susceptible to amino acid substitutions. This discovery paves the way for broadly protective vaccines that are more resistant to viral mutations in HA proteins. Concurrent studies have identified broadly reactive antibodies targeting neuraminidase (NA) and matrix protein 2 (M2) on the viral membrane [19, 20, 21]. Thus, multiple viral antigens have potential target epitopes for broadly protective antibodies. Because these epitopes for broadly protective antibodies are often immunosubdominant in current vaccines, there is a high demand for next‐generation influenza vaccines that can induce broadly protective antibodies robustly and durably [22].

Advances in high‐throughput antibody discovery technologies, such as single B‐cell culture and paired immunoglobulin heavy–light chain sequencing, and in structural biology have substantially advanced our understanding of broadly protective antibody repertoires in humans [23, 24, 25]. Recent findings have revealed how antibody germline genes and their binding modes govern breadth acquisition. They have also helped to explain why broadly protective antibody responses are typically weak following current vaccination regimens. Combining structural and immunological insights, multiple vaccine strategies have been proposed to reshape B‐cell selection and expand on‐target, broad‐spectrum antibody repertoires. In this review, we summarize current knowledge of broadly protective antibodies against influenza viruses, their protective mechanisms, the structural basis of breadth, and the immunological basis for the induction. We also discuss the promises for developing next‐generation vaccines that target the vulnerable sites of viruses.

## 2 Mechanism of Action: How Do Non‐Neutralizing Antibodies Work in Protection?

**FIGURE 1:**
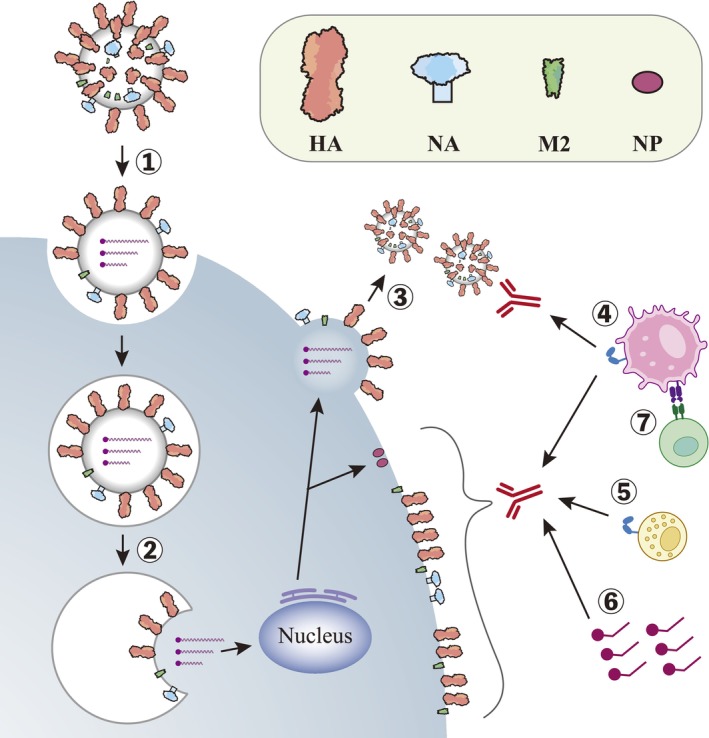
The protective functions of antibodies. Antibodies can inhibit (1) viral attachment and entry, (2) viral fusion and (3) viral budding and release. Furthermore, when bound to viral antigens on the surface of infected cells, antibodies exert multiple protective pathways through their Fc regions, including (4) ADCP (Antibody‐dependent cellular phagocytosis), (5) ADCC (Antibody‐dependent cellular cytotoxicity), (6) CDC (Complement‐dependent cytotoxicity), and (7) CD8^+^ T cell activation.

Neutralizing antibodies serve as the primary arm of vaccine‐induced protective immunity. HAI^+^ antibodies, established surrogate for neutralizing antibodies, are also used as CoP. However, recent studies identified many broadly protective antibodies with little or no neutralization and HAI activity. These broadly protective antibodies activate multiple effector pathways via their IgG Fc regions, leading to the elimination of virus particles and infected cells, independently of neutralization, through immune effector activation. Therefore, understanding these functional aspects is directly relevant to the design of next‐generation vaccines. This chapter reviews the Fc‐dependent protective mechanisms of non‐neutralizing Flu antibodies, focusing on how they cooperate with other immune components (Figure 1).

### 2.1 Fcγ Receptor‐Dependent Pathways

One protective pathway of non‐neutralizing antibodies primarily relies on the interaction between the IgG Fc region and Fc gamma receptors (FcγRs) expressed on the surface of effector cells. The requirement of FcγR‐dependent pathways for in vivo protection has been well established using several models, utilizing Fc‐mutant antibodies with attenuated binding to FcγRs or Fcγ chain‐deficient or FcRα‐null mice [26, 27]. Interestingly, broadly protective HA stalk antibodies activate effector cells via Fc‐FcγR interactions, whereas HAI^+^ HA head antibodies do not [26]. Mechanistically, two independent studies demonstrated that these distinct outcomes are controlled by an additional interaction between sialic acid on effector cells and the HA sialic acid‐binding site on infected cells [28, 29]. Therefore, HAI^+^ HA head antibodies cannot engage this additional interaction because they cover the sialic acid‐binding site of HA through Fab. FcγRs comprise both activating and inhibitory receptors, and the balance of their receptor engagement determines the magnitude of the protective effect [30]. Furthermore, differences in Fc‐linked glycans and FcγR affinity across IgG subclasses also contribute significantly to variability in the magnitude of protective effect [31], even among antibodies targeting the same epitope.

#### 2.1.1 Antibody‐Dependent Cellular Cytotoxicity (ADCC)

The Fc region of IgG is exposed on the surface of infected cells when IgG binds to viral antigens through Fab. Upon Fc recognition on infected cells through FcγR, effector cells initiate antibody‐dependent cellular cytotoxicity (ADCC) [32]. In humans, ADCC is primarily mediated through activating FcγRs, most notably FcγRIIIa (CD16a), which are expressed on the surface of natural killer (NK) cells and, to varying extents, on myeloid cells [32, 33, 34]. Among these effector cells, NK cells are considered to be the principal mediators of ADCC. In the respiratory tract, NK cells comprise both tissue‐resident and circulating subsets, which expand during acute influenza virus infection [35, 36]. Upon Fc engagement, NK cells degranulate and release perforin and granzymes, leading to the programmed cell death of infected cells.

Although animal studies have demonstrated that ADCC activity correlates with the protective function of broadly protective antibodies [37], the overall contribution of ADCC and NK cells to influenza protection remains controversial. Depletion of NK cells through NK1.1 antibody fails to significantly affect the protective activity of non‐neutralizing HA antibodies [38]. Off‐target effects on other NK1.1‐expressing lymphocyte subsets also need to be taken into account in the interpretation of antibody depletion studies [39, 40]. Furthermore, the degree of Fc glycosylation, particularly fucosylation, substantially alters FcγR affinity and ADCC potency [41]. However, the extent of Fc glycosylation in HA monoclonal antibodies has not been fully examined and is likely to be variable among the HA antibody clones. Therefore, the contribution of NK cells to influenza protection is highly context‐dependent and should be interpreted with caution. Conversely, under high‐dose viral challenge in naïve mice, NK‐cell depletion by asialo GM1 antibody can rather improve survival [42], suggesting that NK cells may also contribute to inflammatory pathology in the lung tissue.

#### 2.1.2 Antibody‐Mediated Phagocytosis (ADCP)

Following infection, innate immune cells promptly eliminate pathogens by phagocytosis. Pathogen‐specific antibodies greatly enhance this function by marking targets for uptake, a mechanism termed antibody‐mediated phagocytosis (ADCP), or, classically, antibody opsonization [43]. Effector cells recognize antibody‐coated targets through Fc–FcγR interactions and phagocytose them more efficiently than non‐opsonized targets [44]. In humans, FcγRIIa (CD32a) is the principal phagocytic Fcγ receptor expressed on macrophages and monocytes, while other FcγRs may contribute coordinately depending on cell‐type; these FcγRs collectively drive ADCP effector activity.

Among innate immune cells capable of phagocytosis, macrophages are considered to play major roles in ADCP [38]. During influenza virus infection, alveolar macrophages resident in lung tissue constitute the primary phagocytic population at the site of viral entry. These cells maintain pulmonary homeostasis by clearing apoptotic cells and regulating surfactant turnover [45]. After encountering viruses, alveolar macrophages rapidly internalize antibody‐opsonized viral particles and infected cells, and secrete various cytokines and chemokines that orchestrate subsequent innate and adaptive immune responses [38]. Depletion studies support the essential role of alveolar macrophages in antibody‐mediated protection: intrapulmonary administration of clodronate liposomes, which selectively reduce alveolar macrophage numbers, profoundly diminishes the protective efficacy of non‐neutralizing but broadly protective antibodies in influenza challenge mouse models [38, 46]. Furthermore, the ADCP pathway facilitates antigen presentation, including cross‐presentation, thereby enhancing CD8^+^ T‐cell activation as discussed below. Together, these findings suggest that ADCP mediated by alveolar macrophages provides a mechanistic basis for how non‐neutralizing antibodies can amplify antiviral cellular immunity during the early phase of immune responses following influenza virus infection.

#### 2.1.3 Antibody‐Dependent CD8 ^+^ T Cell Activation

In addition to the Fc‐dependent activation of innate effector cells described above, CD8^+^ T cell‐mediated cytotoxicity is also stimulated by non‐neutralizing antibodies and enhances the ability to eliminate infected cells [46]. In this process, non‐neutralizing antibodies potentiate CD8^+^ T‐cell–mediated protection by modulating alveolar macrophages. Macrophages can process and cross‐present viral antigens more efficiently through ADCP, thereby promoting the robust activation and expansion of virus‐specific CD8^+^ T cells [46, 47]. Additionally, IFN‐γ produced from T cells potentiates alveolar macrophage function and likely creates a positive feedback loop between CD8^+^ T cells and alveolar macrophages [48]. Subsequently, virus‐specific CD8^+^ T cells increase in number, and some differentiate into tissue‐resident memory T cells within the lungs, enabling rapid recall responses at the front line upon secondary exposure [49, 50]. Collectively, non‐neutralizing antibodies enhance the protective function of multiple immune cells, including CD8^+^ T cells, at the local sites of virus infection and contribute to the elimination of infected viruses and the establishment of immunological memory against reinfection.

### 2.2 Complement‐Dependent Pathway

Beyond Fc‐dependent activation of effector cells, antibodies can activate the complement system, including complement‐dependent cytotoxicity (CDC). When antibodies bind to antigens displayed on the surface of infected cells, the classical complement pathway is activated, triggering a proteolytic cascade that results in the formation of the membrane attack complex (MAC) and the lysis of the target cell. While pentameric IgM most efficiently activates the classical pathway, IgG molecules also induce CDC through Fc–Fc–mediated hexamers on the cell surface and facilitate C1q engagement [51]. Fc‐engineered IgG lacking C1q‐binding activity shows reduced protection by non‐neutralizing antibody, indicating that the contribution of the CDC pathway to Fc‐dependent protection [52].

### 2.3 What Are the Target Antigens for Fc‐Mediated Effector Functions?

Although influenza viruses contain multiple antigens capable of eliciting antibody responses, only certain viral proteins serve as effective targets for Fc‐mediated effector functions. Viral envelope proteins HA, NA, and M2 are trafficked to the plasma membrane during viral budding and are then exposed on the surface of infected cells, making them accessible to antibodies [53, 54, 55].

In addition to viral envelope proteins, internal proteins are also expressed on the surface of infected cells. Among them, nucleoprotein (NP) is highly expressed on the infected cells [56, 57, 58]. Several mechanisms have been proposed to account for this unusual surface presentation. One possibility is that NP is trafficked via noncanonical secretory pathways, which transiently deliver internal viral components to the membrane [59]. Another mechanism is the passive acquisition of extracellular NP released from dying or lysed cells, facilitated by its strong self‐oligomerization propensity, which may promote its retention on the bystander cell membrane. These models remain under investigation, and the levels of NP expression may be influenced by both virus strain and cell‐type‐dependent factors.

Despite the expression of NP on infected cells, the protective capacity of anti‐NP antibodies in vivo remains controversial. Some mouse studies demonstrate reduced viral load and morbidity following immunization with recombinant NP or passive transfer of anti‐NP immune serum [60], and anti‐NP B‐cell antigen receptor (BCR) knock‐in mice exhibit substantial resistance to lethal infection by multiple subtypes even in the absence of prior immunization [61]. However, other studies using anti‐NP monoclonal antibodies report minimal or no protective effect against lethal infection in vivo [57, 58]. These discrepancies may reflect differences in antibody epitope specificity, the requirement for particular Fc isotypes, species‐specific expression of surface NP, or variability in effector‐cell engagement. Additional work is needed to determine what conditions are required for NP antibodies to provide heterosubtypic protection.

Efficient Fc‐mediated effector function requires stable formation of an immunological synapse between infected cells and FcγR‐expressing effector cells. HA on infected cells plays a unique role in this process because it can provide additional interactions through HA and sialic acids on effector cells. This HA–sialic acid interaction reinforces target recognition independently of Fc‐FcγR binding and increases the stability of cell–cell conjugates [28, 29]. As a result, HA antibodies benefit from a dual engagement mechanism: epitope recognition plus HA‐mediated cell adhesion, giving them a functional advantage over other antibodies targeting NA, M2e, or NP.

Antigen density and accessibility are also major determinants of Fc‐effector efficiency. HA is shown to be the most abundant viral protein on the plasma membrane of infected cells in vitro, with over 10,000 molecules present per infected cell and a highly exposed ectodomain [62, 63]. NA is typically present at a lower density on the virion; quantitative proteomics showed an approximate HA:NA ratio of 5:1 [64, 65]. While the frequency of NAs on infected cells has not been evaluated, the presence of viral envelope antigens may reflect that of virion NA. M2 is present on infected cells but exposes only a short extracellular domain (M2e) [66], thereby restricting antibody access. NP appears at much lower density than envelope proteins [56], even when present on the cell surface, and may be distributed heterogeneously across the membrane.

Taken together, these structural and quantitative factors strongly suggest that HA is the primary and potent antigenic target for Fc‐mediated effector functions. Its high abundance, large and accessible ectodomain, and ability to mediate sialic acid–dependent adhesion to effector cells collectively enhance Fc‐effector engagement. Consistent with these properties, anti‐HA antibodies generally exhibit stronger ADCC activity than anti‐NA antibodies, reinforcing their central role among non‐neutralizing but protective antibody responses [67, 68].

## 3 Broadly Protective HA Epitopes

**FIGURE 2:**
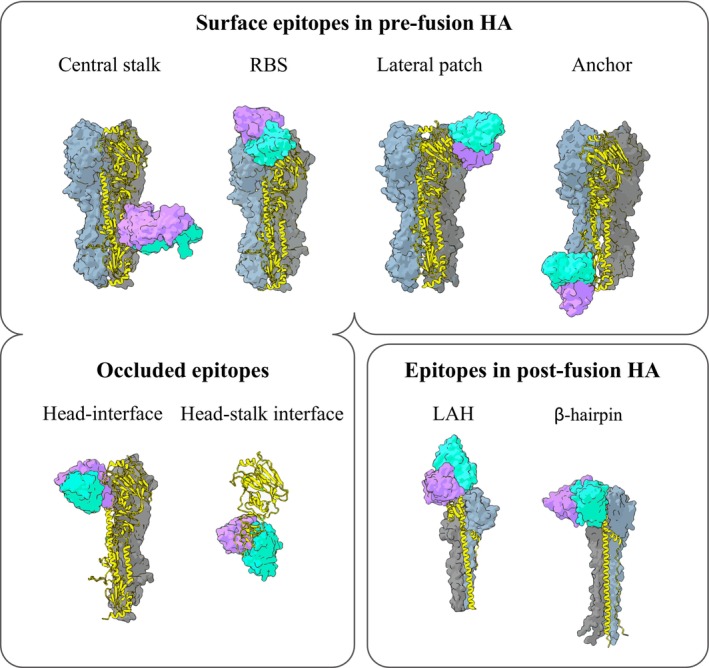
HA epitopes for broadly protective antibodies. The structures of Fab‐HA complexes are presented, including FI6v3 (PDB 3ZTN), K03.28 (PDB 7TRH), Ab6649 (PDB 5W6G), S5V2‐29 (PDB 6E4X), S8V1‐157 (PDB 8US0), and 221‐1C06 (PDB 7T3D). K03.28 and S5V2‐29 were aligned to pre‐fusion HA (PDB 3ZTN). The post‐fusion HA antibodies are also presented, including S1V2‐72 (PDB 8UDG) and LAH31 (PDB 8IB1). LAH31 was aligned to post‐fusion HA (PDB 1HTM). The Fab heavy and light chains are colored purple and green, respectively.

Given the increasing interest in broadly protective HA antibodies against antigenically distinct influenza viruses, many researchers have enthusiastically pursued the isolation and characterization of potent antibody clones for several decades. Significant advances have been made in the field by linking HA structural constraints to the genetic basis of antibodies. Notably, identifying evolutionarily conserved HA epitopes and analyzing the genetic and structural features of on‐target antibodies that recognize them reveals the mechanisms underlying the broad protection (Figure 2).

### 3.1 Central Stalk (CS) Epitope

One of the best‐characterized epitopes targeted by broadly protective antibodies is the conserved epitope located in the HA stalk region of the HA2 domain, called “Central stalk (CS) epitope”. This epitope region exhibits low structural mobility and strong functional constraints on viral fusion processes, reducing the likelihood of antigenic mutation. Consequently, antibodies targeting this functionally restricted and conserved region often exhibit broad neutralization activity across intra‐group (Groups 1 and 2) or inter‐group subtypes of influenza A viruses. A few antibodies also show activity across influenza A and B [69, 70, 71, 72, 73, 74, 75, 76].

The CS epitope is highly conserved across different influenza virus strains due to strong structural constraints. This CS region contains key components involved in viral membrane fusion, including the α‐helix bundle of the HA2 subunit, the fusion peptide proximal regions, and the hinge region. These structures are essential for the viral replication cycle, thereby limiting tolerance to amino acid substitutions. While antibodies targeting the CS region often show broad reactivity, there are substantial levels of variation in the binding modes. Some antibodies, such as CR6261, insert hydrophobic residues encoded within HCDR2 into a conserved hydrophobic groove at the HA1–HA2 interface and engage the epitope predominantly through its heavy chains [69, 70]. In contrast, FI6 differs from this class of antibodies in that it recognizes a broader region spanning both HA1 and HA2 and utilizes a distinct binding orientation, achieving inter‐group neutralization [71]. Furthermore, CR9114 has been shown to access a highly conserved region shared by influenza A and B, thereby achieving near‐pan‐influenza breadth [73].

The genetic basis of this antibody repertoire exhibits striking convergence in the utilization of specific immunoglobulin heavy‐chain V (IGHV) gene families, particularly IGHV1‐69. The presence of the germline‐encoded hydrophobic residues in HCDR2 encoded by IGHV1‐69 is essential for binding to the hydrophobic patch in the stalk region [69, 70, 72, 73]. Additionally, the presence of a specific amino acid motif in HCDR2 has been associated with enhanced binding ability and breadth [71, 73, 77]. Although generally less restrictive than those imposed on the heavy‐chain, light‐chain constraints also contribute to antigen‐binding, and particular light‐chain configurations have been shown to influence the broad reactivity [71, 72, 73]. Aside from IGHV1‐69 antibodies, IGHV6‐1 and IGHV1‐18 clonotypes have also been reported in these repertoires [74, 75, 78]. Interestingly, many of these antibodies often achieve high affinity with relatively few somatic hypermutations. Thus, it is conceivable that the genetic templates of CS‐directed antibodies targeting vulnerable sites of viruses may have been evolutionarily favored in the human antibody repertoire.

### 3.2 Receptor‐Binding Site (RBS) Epitope

Influenza virus establishes host attachment and entry via interactions between the receptor‐binding site (RBS) in the head region and sialic acids on the host cell surface. Thus, the head antibody masking RBS can exhibit potent neutralization activity and HAI activity. However, many antibodies targeting the head domain exhibit specificity to antigenically homologous strains only because they recognize the highly variable region surrounding the RBS. In contrast, the RBS is structurally critical for viral infection, and the overall architecture is highly conserved across the same subtypes of influenza A and B. Antibodies targeting the RBS epitope acquire broad, typically intra‐subtype, neutralization activity [79, 80, 81, 82, 83, 84, 85, 86, 87]. Furthermore, some studies have found that a class of antibodies exhibits inter‐group neutralization activity against H1 and H3 strains [24, 88, 89].

These RBS clones tend to use unique binding modes for targeting the epitopes. The RBS in HA forms a shallow pocket structure that engages sialic acid for binding to the host cell. Most RBS‐directed antibodies do not exhibit broad activity due to their large footprints, which simultaneously recognize variable residues surrounding RBS [86]. However, a small number of broadly reactive RBS‐directed antibodies insert their HCDR3 loop into the receptor‐binding pocket by mimicking the key contact site of sialic acid with the receptor‐binding site [81, 83, 84, 85]. This HCDR3 insertion provides strong affinity with fewer additional contacts to variable rim residues and enables broad reactivity due to minimal peripheral contacts. Furthermore, some of the RBS‐directed antibody clones enhance neutralization activity against different subtypes by increasing binding avidity through bivalent inter‐HA interactions [82, 84].

V_H_ gene usage in this antibody repertoire is relatively heterogeneous without strongly relying on a single germline [85, 90, 91], as contrasted with other broadly protective antibody repertoires described below. An exception is RBS antibodies against influenza B viruses that use particular V_H_ genes [87]. Despite the heterogeneity in V_H_ usage, HCDR3 of broadly reactive RBS antibodies is often structurally convergent, characterized by relatively long lengths and shared key acidic residues, features that represent sialic acid mimicking geometries [24, 85].

### 3.3 Lateral Patch Epitope

In addition to the RBS, several conserved epitopes have been identified in the HA head domain. One is the “Lateral patch” epitope, the β‐sheet region spanning from the rim of the RBS to the lateral side of the head domain [91, 92, 93]. As a surface‐exposed region of the HA head, the lateral patch would be expected to be susceptible to antigenic drift. However, in the seasonal H1N1 viruses, the major residues of the lateral patch remained relatively stable for several decades until the emergence of the H1N1 pandemic [94, 95]. Moreover, this epitope remained highly conserved across many human and zoonotic strains across diverse H1 lineages.

Lateral patch antibodies are generally categorized into two structural classes based on their binding geometry [94]. One class of antibodies approaches the HA head from a perpendicular angle. They often utilize a recurring Y‐x‐R/K motif within their HCDR3 to make critical contacts with the lateral patch. Another class of antibodies targets the upper portion of the lateral patch and partially overlaps the RBS‐adjacent region. Their binding can be sensitive to specific mutations, which accumulated during the antigenic drift of pandemic H1N1 strains, highlighting the impact of viral evolution on lateral patch‐directed antibodies.

### 3.4 Head‐Interface Epitope

A new class of epitope was identified at the head–head interface of the trimeric HA structure. This epitope is quite distinct from previous epitopes; it is buried inside the trimer interface of pre‐fusion HA. This head‐interface epitope is a conserved site located at the contact sites between neighboring HA protomers. Under the pre‐fusion HA structure, the epitope is structurally hidden from antibody access [96, 97]. Although the antibodies cannot access the head‐interface epitope in the closed pre‐fusion trimer due to steric hindrance, it is postulated that the HA trimer structure is more flexible than previously considered, undergoing dynamic changes denoted as the molecular “breathing” of HA trimer, in which HA trimers open up the trimeric head structure in a way to temporarily expose the internal epitopes [98].

Molecular breathing has also been reported in the envelope proteins of human immunodeficiency virus‐1 (HIV‐1) and dengue virus [99, 100, 101, 102]. Antibodies targeting this epitope exhibit potent, broad protective activity across Groups 1 and 2. This class of antibody does not exhibit detectable neutralization activity. However, in addition to the Fc‐mediated protective functions, it also prevents viral cell‐to‐cell spread, which may be due to disrupting the HA trimer's conformation by binding [97].

Head‐interface antibodies are heterogeneous in the V_H_ gene usage [103, 104]. Similarly, they also utilize a variety of V_L_ genes. However, a public clonotype utilizing IGκV1‐39 has been reported. This is consistent with the structural characteristics of epitopes, which rely on main‐chain‐driven interactions and form conserved surfaces with low side‐chain specificity. A variety of V_H_/V_L_ germlines likely adopt the binding modes in a compatible way. Conversely, many antibodies have been shown to possess relatively high levels of somatic hypermutation. Recurrent convergent mutations are inserted into CDR H2/H3 or light chain CDR1/2, stabilizing and optimizing the antibody binding angle. Therefore, while these antibodies utilize diverse germline template genes, they require structural optimization through somatic hypermutations for acquiring high affinity to head‐interface targeting.

### 3.5 Head‐Stalk Interface Epitope

The head‐stalk interface epitope is characterized as a conserved structural epitope located at the interface between the HA head and stalk regions [105]. This is the region where the head domain and stalk domain normally come into close contact within the trimer structure. Similar to the head‐interface epitope, this head‐stalk interface epitope is largely concealed in the pre‐fusion HA trimer. Again, however, the viral breathing motion of the HA trimer is suggested to enable the epitope exposure and antibody access. This head‐stalk interface region is conserved across many influenza A subtypes and is recognized as a conformational epitope because it maintains a continuous three‐dimensional arrangement upon antibody binding. Although this antibody class does not exhibit neutralizing activity in vitro, it confers protection in mouse infection models and binds broadly to influenza A subtypes spanning Groups 1 and 2. The antibodies targeting this epitope exhibit a bias toward specific V_L_, but do not show a strict restriction in V_H_/V_L_ gene usage.

### 3.6 HA Anchor Epitope

HA anchor epitope exists in the HA stalk at the most proximal region to the viral membrane [25]. It is characterized by a highly conserved structural region at the base of the HA stalk. The HA anchor epitope is typically occluded from B‐cell recognition due to the proximity between the epitope and the viral membrane. Despite its membrane‐proximal location, this epitope might be temporarily exposed due to the flexibility of the HA trimer [106]. Antibodies targeting this epitope exhibit broad neutralization activity against H1N1 strains; in some cases, they cross‐react with other Group 1 HA subtypes, such as H2 and H5.

Anchor epitope‐targeting antibodies utilize highly restricted V_H_3/V_K_3 genes and share a common binding motif, such as a specific sequence in the CDR3 of the κ‐chain [25, 107]. These convergences likely reflect the conservation of the anchor epitope, as well as the shared conformational properties and conserved contact residues for antibody binding. Unlike typical stalk‐targeting CS antibodies, HA anchor epitope antibodies commonly employ an upward approach angle, due to targeting the epitope near the membrane from above.

### 3.7 Post‐Fusion HA Epitopes

Some of the broadly protective epitopes mentioned above are located in regions that are normally shielded or structurally inaccessible to antibodies on the pre‐fusion HA trimer on the virus particle. Recent studies have identified novel epitopes that are shielded in the native pre‐fusion HA state but exposed in the post‐fusion HA state, which arises from dramatic conformational changes at low pH during the virus infection cycle [108].

One of the epitopes specific in the post‐fusion HA state is the long α‐helix (LAH) epitope. The α‐helical region in the HA stalk is located near the internal core and contributes to the stabilization of the trimeric base structure of HA [109]. Because this region forms a fundamental structural element of the stalk region, its sequence and conformation are highly conserved. However, the LAH epitope is structurally occluded in the pre‐fusion HA state and becomes exposed during the transition to the post‐fusion HA state. Early work nevertheless identified a broadly protective monoclonal antibody in mice that targets this region, and epitope mapping localized its binding site to the HA2 domain [110]. Owing to its high conservation and continuous helical structure, the LAH region has been considered a promising candidate for epitope‐focused peptide vaccine design [111].

More recently, several human monoclonal antibodies directed against LAH have been isolated, and these demonstrate broad in vivo protection against Group 2 influenza A viruses, including H3N2 and H7N9 [109]. Among LAH‐directed antibodies in humans, LAH31 has notable breadth covering the Group 1 and 2 HA subtypes [52]. Structural analysis reveals that LAH31 recognizes an epitope that is exposed only in the post‐fusion state and not in the native pre‐fusion conformation. Upon pH‐triggered conformational changes, this epitope region dramatically changes the structure from an α‐helix to a loop, thereby creating the neo‐epitope structure in the post‐fusion state.

A recent study further identified a class of antibodies that target the highly conserved epitope structure that remains unchanged during the transition from pre‐fusion to post‐fusion HA. This “β‐hairpin epitope” refers to a β‐hairpin structure located in the terminal site of the HA2 domain [89]. In the pre‐fusion HA, this structure is part of the β‐sheet formed by residues of the HA1 and HA2 domains. Although the β‐hairpin epitope is difficult to access in the statically stable, native pre‐fusion HA structure, the β‐hairpin‐directed antibody, S1V2–72, can exhibit binding ability to recombinant pre‐fusion HA and HA‐expressing cell line. Temporary exposure due to molecular fluctuations might allow antibody binding to this epitope, like the head‐interface antibodies. Conversely, the β‐hairpin epitope exists stably as an isolated loop in the post‐fusion HA structure, characterized by a reversed hairpin comprising two short β‐strands. Because its epitope is highly conserved at the primary sequence level, S1V2–72 exhibits broad‐spectrum protection against influenza A and influenza B strains.

How HA on the infected cell surface becomes a target of post‐fusion HA‐directed antibodies is not fully understood. Post‐fusion HA is generated only under acidic conditions (pH < 5.6) within host endosomes during viral fusion [108], and is therefore not typically present in the extracellular milieu. Nevertheless, LAH‐directed antibodies bind to infected cells with decent binding affinity and mediate in vitro ADCC activity [52, 55]. It is also noteworthy that conformation‐sensitive CS epitope antibodies bind to infected cells, suggesting that HA on the infected cell surface is structurally heterogeneous, comprising native pre‐fusion HA intermingled with post‐fusion HA or intermediate states that expose normally occluded LAH epitope while preserving CS epitope. In any case, sites of acidic exposure within infected cells could generate non‐native HA conformations with an exposure of LAH‐like epitopes.

One possible mechanism is a drop in intracellular pH within dying cells [112, 113]. Influenza infection triggers programmed cell death, including apoptosis and necroptosis, in epithelial and immune cells [114]. In vitro studies have reported a decrease in intracellular pH within a few hours after the onset of apoptosis [115, 116, 117]. Although the degree of acidification varies with the induction method and time point, intracellular pH commonly declines to approximately 5.6–6.6 in these contexts. Furthermore, during cell death, including necroptosis, events such as lysosomal membrane permeabilization and ATP depletion can further decrease intracellular pH [118, 119]. Therefore, dying cells may create local acidic environments that drive HA into non‐native conformations.

A related question concerns the structural state of HA on the infected cell surface. HA is synthesized in the endoplasmic reticulum (ER) as HA0, the uncleaved precursor that cannot undergo full transition to the post‐fusion state, and is transported to the plasma membrane via the Golgi complex and the trans‐Golgi network (TGN) [120]. HA0 is cleaved by host proteases such as human airway trypsin‐like protease (HAT) or transmembrane protease serine S1 member 2/4 (TMPRSS2/4) either during secretory trafficking or at the plasma membrane [121, 122], except for the highly pathogenic avian influenza H5 and H7 viruses, which have a furin cleavage site [123]. These trafficking and processing steps raise questions about where and when HA might be exposed to sufficiently acidic conditions to induce structural change.

In this context, two scenarios can be envisaged. First, intracellular cleavage by TMPRSSs, followed by exposure to low pH in the TGN, could induce a conformational change toward a post‐fusion HA. However, luminal pH in the TGN is generally maintained near pH 6.0, and the influenza M2 proton channel prevents premature HA conformational change during trafficking [124]. Under conditions of cellular stress and loss of organelle integrity during cell death, however, TGN pH homeostasis could collapse, allowing luminal acidification below typical levels. In this scenario, HA deposited at various stages of the cell death process could exist in multiple conformations on the plasma membrane, which may help account for observations made in vivo but cannot be adequately reproduced in vitro, particularly in MDCK cells that lack TMPRSS expression [125].

Second, although HA0 is less sensitive to low pH than cleaved HA [126], it nonetheless undergoes substantial conformational changes at acidic pH [127]. Acid‐treated HA0 adopts a structure distinct from canonical post‐fusion HA, characterized by an open head interface, partial extension of the α‐helical region, and relocation of the fusion peptide. While this conformational change is reversible, the exposure of internal hydrophobic regions may promote aggregation or interaction with other proteins, stabilizing the non‐native HA conformation. Moreover, acid‐induced structural rearrangements could expose normally occluded protease cleavage sites, potentially permitting cleavage events that would not occur under neutral conditions.

In summary, the heterogeneity of HA antigen structures displayed on infected cells and the mechanisms by which these variants arise have not yet been fully elucidated. A more comprehensive understanding of these processes will be essential for identifying attributes of HA that could be exploited in next‐generation vaccine design.

## 4 Why Are Broadly Protective Antibody Responses Rare?

**FIGURE 3:**
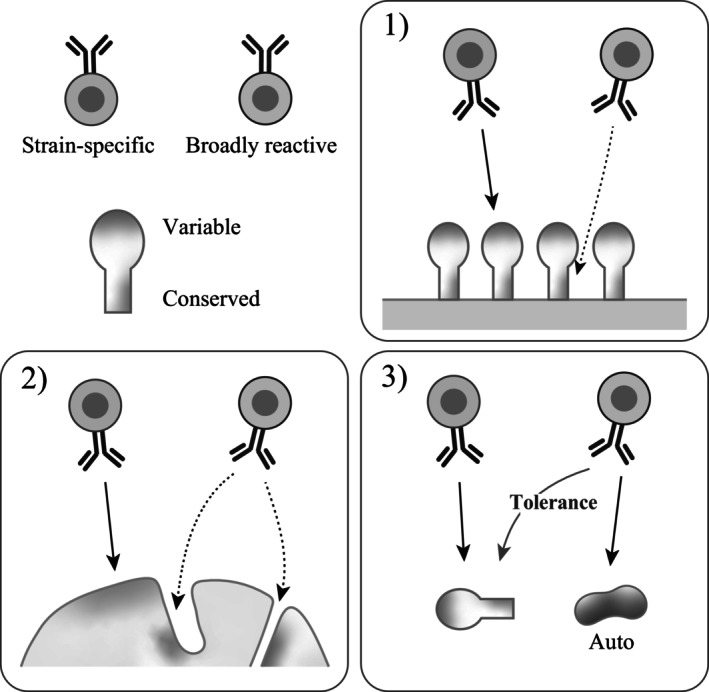
Conceptual scenarios of how broadly protective epitopes are concealed and why they became immunosubdominant. Poor accessibility due to (1) steric hindrance by the variable head region and (2) the occluded nature of epitopes. (3) Immune tolerance due to autoreactivity.

Neither vaccination nor infection elicits B‐cell responses with dominance toward the epitopes for broadly protective antibodies as described above. Instead, antibody repertoires are shaped by a cellular hierarchy known as immunodominance, whereby head epitopes for neutralizing antibodies outcompete others under the current vaccination or natural infection. Although broadly protective epitopes do exist in conventional vaccine antigens, they usually fail to elicit strong antibody responses. One major explanation is that these epitopes are physically or functionally hidden from B cell and antibody access. The following conceptual scenarios illustrate how such epitope concealment arises and why it impairs the generation of broadly protective antibodies (Figure 3).

Germinal centers (GCs) impose stringent Darwinian selection on B cells [128]. After diversifying B cell antigen receptors (BCRs) through somatic hypermutation in their variable regions, B cell clones are selected based on the competition for limited antigen and T‐cell help. Only those displaying higher‐affinity BCRs can successfully extract antigen from follicular dendritic cells (FDCs) and receive survival and differentiation signals from T follicular helper (Tfh) cells [129]. Since membrane‐bound BCRs exhibit constrained mobility and a fixed orientation, their antigen‐binding affinity is particularly susceptible to the accessibility to the epitope due to the steric hindrance. Thus, B cell clones suffer from a great disadvantage during this competition when they recognize occluded or otherwise geometrically restricted epitopes. Even if these epitopes can be targeted by soluble antibodies, B cells against occluded or geometrically restricted epitopes are outcompeted by clones recognizing more accessible epitopes. This competitive disadvantage strongly biases GC selection toward immunodominant epitopes and away from subdominant ones.

The organization of HA trimers on virus particles further restricts access to some conserved epitopes. The HA stalk region is positioned proximally to the viral membrane and is shielded by the overhanging HA head “canopy.” This steric arrangement limits BCR penetration into the stalk region. Consistent with this, HA stalk‐directed antibodies exhibit reduced binding capacity to virus‐anchored HA relative to soluble HA, whereas HA head‐directed antibodies bind both forms equivalently [130]. Moreover, because BCRs are anchored to the plasma membrane, their approach angles are more constrained relative to soluble IgG antibodies. This geometric limitation might reduce the likelihood that B cells can productively engage membrane‐proximal or lateral stalk epitopes on membrane‐anchored HA, such as CS and anchor epitopes. Importantly, conventional split‐virion seasonal vaccines largely preserve the native orientation of HA, reinforcing B cell recognition toward the membrane‐distal head epitope and away from the membrane‐proximal conserved stalk epitopes [130, 131].

The architecture of HA itself poses a key challenge to the development of broadly protective B cell responses. Although the HA head region contains several conserved epitopes capable of mediating broad protection, many of these are partially buried within the trimer or recessed in space. The RBS epitope, located at the upper part of the head, forms a narrow, pocket‐like recess flanked by hypervariable loops [24, 79, 80, 81, 82, 83, 84, 85, 86, 87, 88, 89]. This topology attenuates BCR access and restricts productive engagement. Furthermore, the head‐interface epitope lies at the junction between adjacent HA monomers. This epitope is largely occluded in the native pre‐fusion HA conformation and is presumably exposed during molecular fluctuations [96, 97], making it a poor target for B cells. Additionally, post‐fusion epitopes, such as LAH, are more inaccessible under the pre‐fusion HA structure, even with molecular fluctuations [52]; therefore, the immune system cannot recognize these invisible epitopes in a typical setting. In each case, the structural topology favors immunodominant, surface‐exposed epitopes and marginalizes conserved epitopes, despite their functional relevance.

An additional layer of constraint arises from the binding properties of some of these antibodies. Several human monoclonal antibodies targeting HA stalk show polyreactivity to self‐antigens, including LPS, insulin, and double‐stranded DNA [130, 132, 133]. If some epitopes structurally resemble host molecules, B cells that develop and express BCRs specific for these epitopes may be eliminated or functionally silenced during central or peripheral tolerance checkpoints. This molecular mimicry provides a possible mechanism by which viruses exploit host tolerance systems to evade broadly protective antibodies against not only influenza virus but also HIV [134, 135]. In this context, HA stalk antibodies show higher polyreactivity than HAI^+^ HA head antibodies [130]. Additionally, such polyreactive properties are observed among antibodies targeting the RBS and the lateral patch epitopes [132, 133], indicating that polyreactivity is a quite common feature across different classes of broadly protective antibodies. While the extent to which polyreactivity suppresses B cell development is debatable, immune tolerance narrows the diversity of the peripheral antibody repertoire [136]. Furthermore, the induction of broadly reactive Flu antibodies has been shown to increase serum polyreactivity and susceptibility to autoimmunity in mouse models [137], implying that self‐tolerance continuously suppresses broadly protective humoral responses. In line with this, modulating immune checkpoints during HIV‐1 vaccination facilitates the selection of envelope protein‐reactive B cells in mice and macaque models [138].

These structural, positional, and immunological constraints collectively create a landscape in which conserved, broadly protective epitopes are systematically outcompeted by immunodominant, variable epitopes. Influenza virus appears to have evolved geometries and molecular surfaces that minimize exposure of its most vulnerable sites. Consequently, standard vaccination rarely elicits antibodies that target these conserved regions, even though they are present within the antigen. Understanding these principles may provide a mechanistic foundation for rational vaccine design strategies that aim to override immunodominance and redirect B cell responses toward protective subdominant epitopes.

## 5 Elicitation of Broadly Protective Antibody Responses

The multiple layers of constraints within HA make conserved epitopes immunosubdominant, leading to broadly protective antibody responses being typically rare in routine vaccination. However, there are situations where broadly protective antibody responses are enhanced. For example, serological analyses have shown that natural infection rather than vaccination preferentially induces broadly reactive flu antibodies in the blood of humans and mice [139, 140]. These findings suggest that natural infection may induce humoral immune responses with a distinct epitope landscape, presumably due to the infection‐dependent factors. In line with this, enhanced broadly reactive antibody responses have been established in cases of infection with pandemic viruses [141, 142]. In this chapter, we discuss natural settings that induce broadly protective antibody responses, their immunological basis, and the implications for developing next‐generation vaccines.

### 5.1 Humoral Immune Responses at the Site of Viral Replication

The humoral immune response is typically initiated in secondary lymphoid organs such as the lymph nodes and spleen when the antigens are immunized through systemic routes. However, humoral immunity is not only confined to these secondary lymphoid organs; it can also develop tertiary lymphoid organs in non‐lymphoid tissues [143, 144]. In the context of respiratory diseases, including those caused by viral or bacterial infections, as well as conditions such as smoking, allergy, and autoimmunity, inducible bronchus‐associated lymphoid tissue (iBALT) can form in the lung [145, 146]. In secondary lymphoid organs, lymphocytes are organized into highly structured areas, such as B‐cell follicles and T‐cell zones, which are clearly separated by distinct stromal cells and follicular dendritic cell networks [144]. While the structure of iBALT is similar to that of secondary lymphoid organs, several groups have reported unclear boundaries between B‐cell follicles and T‐cell zones, with low density of GC B cells [147, 148]. Interestingly, mice that form iBALT but lack secondary lymphoid tissue do not lose resistance to infection, underscoring the important role of iBALT and the ectopic humoral immune responses in defending the host against respiratory viral infections [149, 150].

Although iBALT exhibits histological differences from secondary lymphoid organs, lung GC B cells undergo class‐switch recombination and accumulate somatic hypermutations, indicating that pulmonary GC responses are functionally comparable to those in conventional secondary lymphoid tissues [147, 151, 152]. One notable feature of lung GCs is their delayed kinetics. Whereas GC responses in secondary lymphoid organs reach a plateau approximately 10 days after infection, GC formation in the lung continues to increase and reaches a plateau only around day 20 [147]. Despite this delayed onset, lung GCs persist substantially longer than their counterparts in secondary lymphoid organs [147]. Because B‐cell clonal expansion and antigen persistence are key determinants of GC maintenance [153], these observations suggest that the pulmonary environment supports prolonged GC activity. Consistent with this idea, lung GC B cells display a more proliferative phenotype than GC B cells in secondary lymphoid organs [147]. Furthermore, viral RNA remains detectable in the lung for up to 1 month after infection, suggesting that persistent pulmonary GC responses may be sustained by continued antigen availability even after viral clearance [154].

A unique feature of these persistent lung GCs is the preferential selection of broadly reactive B‐cell repertoires. Cross‐reactivity against both homologous H3 strains and antigenically divergent influenza viruses is significantly higher among lung GC B cells than among their counterparts in secondary lymphoid organs following infection [147]. Epitope mapping of monoclonal antibodies generated from single‐cell cultures of lung GC B cells revealed that approximately 50% of cross‐reactive HA‐binding clones targeted the LAH epitope, whereas smaller fractions recognized HA head‐ and CS epitope‐directed antibodies [109]. Consistent with the structurally occluded nature of the LAH epitope, native pre‐fusion HA probes detected only approximately 20% of HA‐binding GC B cells identified using monomeric HA probes in the lung. Furthermore, cross‐reactive GC B‐cell responses are not efficiently induced following intranasal immunization with inactivated influenza vaccines [109]. Together, these findings suggest that HA antigens that expose otherwise hidden epitopes are presented within lung GCs and that this phenomenon is associated with live‐virus infection rather than merely with the route of antigen administration.

Following GC responses, memory B cells persist within the infected lung for several months [147, 151, 155, 156]. These lung‐resident memory B cells (BRMs) express tissue‐retention markers, including CXCR3, CCR6, and CD69, which contribute to their localization and long‐term maintenance within the lung [151, 156]. Notably, the cross‐reactivity of the pulmonary BRM compartment increases sharply after the establishment of robust lung GC responses [147]. Conditional deletion of Bcl‐6 during this period reduces cross‐reactivity within the lung memory B‐cell pool while leaving the splenic memory compartment largely unaffected. Furthermore, fate‐mapping studies using intratracheal EdU labeling support the conclusion that lung GCs preferentially select cross‐reactive B‐cell repertoires and subsequently generate long‐lived resident memory B cells in the lung.

**FIGURE 4:**
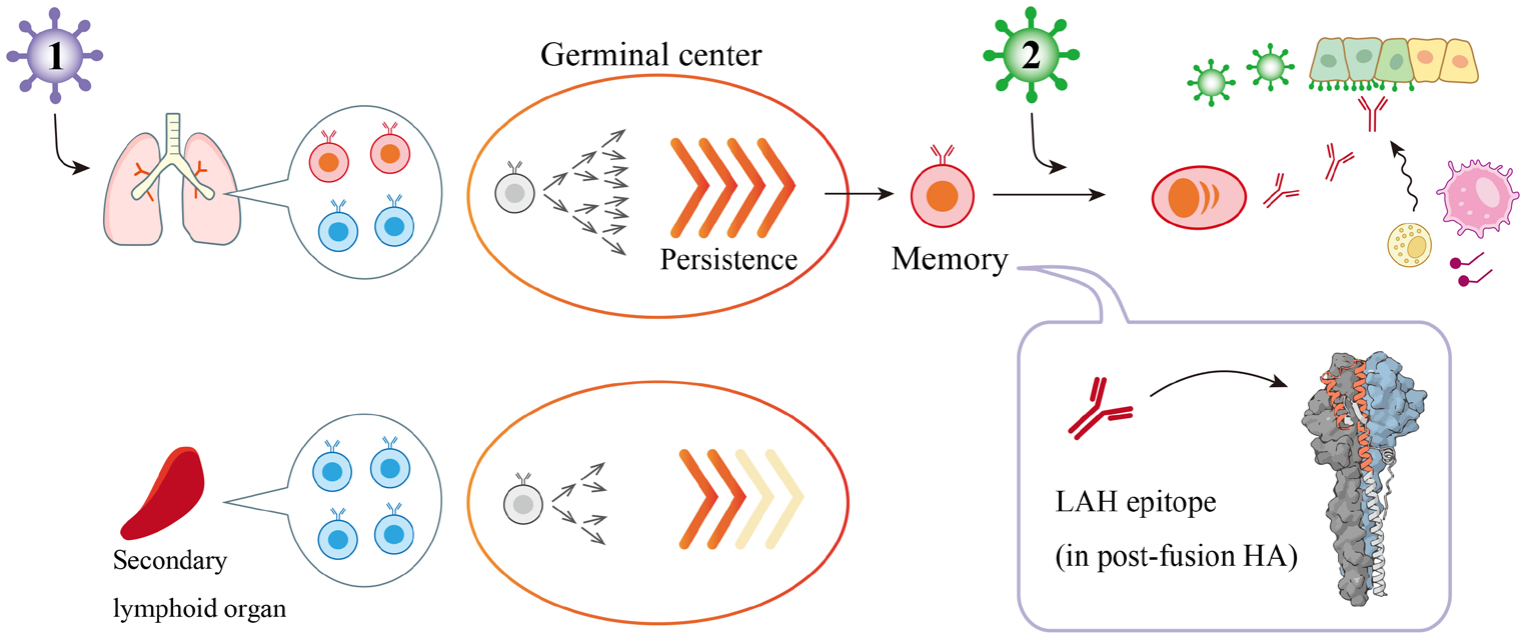
Broadly protective humoral responses at the site of infection. The LAH epitope (red) is highlighted on the post‐fusion HA trimer (PDB 1HTM).

Upon secondary infection, BRMs rapidly proliferate and differentiate into antibody‐secreting cells, thereby providing high local concentrations of protective antibodies at the site of pathogen entry [156]. Interestingly, memory B cells isolated from human lungs exhibit transcriptional profiles that resemble those of murine lung BRMs [155]. Collectively, these findings support the concept that local humoral immune responses generated at the site of infection contribute to broad protection against subsequent infection by antigenically divergent viruses (Figure 4).

A key unresolved question is how lung GCs present antigens capable of selecting these broadly reactive B‐cell repertoires. The specialized antigen‐presenting cells within GCs are FDCs, and a CD35 (CR1)^+^ stromal network resembling FDCs has also been identified in influenza‐induced iBALT [148]. How these cells present epitopes that are normally inaccessible on native HA remains unclear. One possibility is the capture and retention of HA‐containing debris released from dying infected cells, analogous to the presentation of internal NP antigens. As discussed in Chapter 3, HA molecules in non‐native conformations recognized by LAH‐directed antibodies can be detected on the surface of infected cells. A second possibility is that transient exposure to acidic environments during antigen processing and recycling contributes to the generation or preservation of such conformations [157]. Although recycling pathways that return cargo to the plasma membrane generally avoid strongly acidic compartments and are typically restricted to environments above approximately pH 6.0, infection‐induced alterations in the lung microenvironment may permit alternative trafficking routes that expose antigens to lower pH conditions. While direct evidence for these mechanisms is currently lacking, they could provide a source of structurally altered HA antigens that promote the preferential selection of LAH‐reactive B cells within lung GCs.

The above findings suggest that viral antigens may undergo conformational changes or biochemical modifications in vivo. This results in structurally altered antigens that expose epitopes absent from the native structure; those antigens are often referred to as “dark antigens” [158]. Such phenomena may not be unique to influenza virus infection. Indeed, flow cytometric analyses have identified human B cells that recognize the respiratory syncytial virus (RSV) fusion (F) antigen in its post‐fusion conformation [159, 160], suggesting that B cell responses to non‐native viral antigens may occur in multiple viral infections. Notably, similar patterns of heterogeneous B‐cell selection have also been observed following systemic immunization [161], suggesting that the generation of structurally altered antigens is not unique to viral infection and may represent a more general feature of immune responses to viral antigens. Despite this, during natural influenza infection, the generation of structurally altered viral antigens may broaden the selection of protective B cell repertoires by exposing conserved but occluded epitopes, such as the LAH epitope, that are normally inaccessible in native viral antigen structure.

### 5.2 Pandemic H1N1 Virus Infection

Another real‐world example of an enhanced cross‐reactive antibody response was observed during the 2009 H1N1 pandemic (H1N1pdm09). The H1N1pdm virus emerged in 2009 through a series of complex reassortment events among swine influenza viruses, ultimately causing a global pandemic [162, 163]. The appearance of this novel virus prompted extensive analyses of humoral immune responses in infected individuals, as well as clinical trials of newly developed subtype‐specific vaccines, generating a substantial body of data. Because neutralizing HA antibodies induced by prior seasonal H1N1 infections or vaccinations showed minimal cross‐reactivity to H1N1pdm09 due to its pronounced antigenic divergence, it was initially assumed that the inactivated H1N1pdm09 vaccine would require two doses, potentially with an adjuvant, to achieve sufficient immunogenicity. Contrary to these expectations, however, clinical trial data revealed that even a single dose of the non‐adjuvanted split vaccine‐elicited robust HAI (and neutralizing) antibody responses, providing seroprotection in most vaccine recipients [164].

Further repertoire analyses in individuals vaccinated or infected with H1N1pdm09 revealed that monoclonal antibodies derived from plasmablasts target conserved epitopes on both the HA head and stalk, exhibiting broad neutralizing activity against seasonal H1N1 and H5N1 strains [141, 165]. Notably, these cross‐neutralizing antibodies display high levels of somatic hypermutation in their immunoglobulin genes, indicating that they originate from memory B cells formed during prior influenza exposures. Consistent with this notion, H1N1pdm09‐reactive memory B cells are detectable even before vaccination [165]. These findings demonstrate that boosting with antigenically divergent antigens preferentially reactivates pre‐existing cross‐reactive memory B cells rather than eliciting *de novo* responses from naïve B cells to newly encountered epitopes. Although conserved epitopes can become immunosubdominant due to the constraints discussed in Chapter 4, prior antigenic exposures can shape both the quality and quantity of subsequent B‐cell responses and can alter B‐cell hierarchy, a phenomenon referred to as “immune imprinting” [166, 167].

Overall, the evidence suggests that, under specific immunological conditions, the inhibitory mechanisms that usually constrain antibody responses can be overcome, thereby increasing the likelihood of generating broadly protective antibody responses. These findings provide important clues for next‐generation vaccine strategies aimed at selectively eliciting antibody responses against target epitopes that are ordinarily immunosubdominant.

## 6 Toward Next‐Generation Influenza Vaccines

**FIGURE 5:**
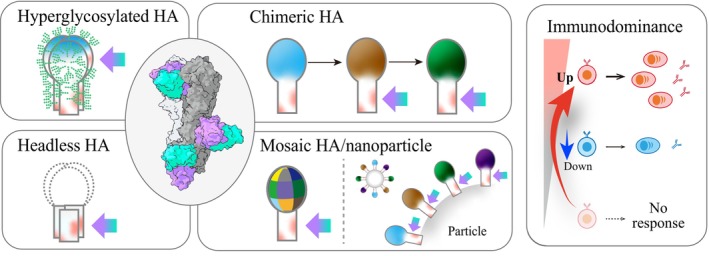
Epitope‐focused vaccine strategies. Three Fab‐HA complexes were aligned to the pre‐fusion HA trimer (PDB 3ZTN), including FI6v3 (PDB 3ZTN), 221‐1C06 (PDB 7T3D), and S5V2‐29 (PDB 6E4X).

Extensive immunological and serological analyses have clarified the structural features of broadly protective antibodies and their epitopes, the determinants on immunodominance, and the specific conditions under which these restrictions can be overcome. Based on this accumulating knowledge, multiple rational vaccine designs and vaccination strategies that dictate humoral immune responses toward target epitopes have been proposed (Figure 5).

The earlier identification of broadly protective epitopes in the HA stalk region has led to the development of designed vaccines that elicit antibody responses to those epitopes. The “headless HA” antigen, which involves removing the HA head domain to eliminate steric hindrance as discussed in Chapter 4, has been explored for decades [168, 169, 170]. However, maintaining the structural stability of the native stalk conformation had posed a significant challenge; two studies address this issue and provide a roadmap for subsequent developments [171, 172]. The sequential vaccination strategy of “chimeric HA”, a prime‐boost regimen involving HAs that share an identical stalk domain with antigenically distinct “exotic” head domains [173], is conceptually supported by observations from H1N1pdm09, as discussed in Chapter 5. These vaccine strategies effectively elicit antibody responses targeting stalk epitopes in humans, including the CS and anchor epitopes [25, 174, 175, 176, 177].

Other strategies include the usage of hyperglycosylated HA that can mask immunodominant regions from B‐cell recognition through introducing additional N‐linked glycans and altering the immunodominance hierarchy. This strategy succeeded in redirecting antibody responses toward HA stalk epitopes [178] or the head‐interface epitope [179]. Other approaches consistently utilize mosaic HA designs, but differ in the ways of antigen presentation and immunization. One involves sequential vaccination with mosaic HA antigens, patchworks of variable head antigenic sites derived from exotic strains [180, 181], an approach that is conceptually similar to chimeric HA. The other involves mosaic nanoparticles, which display multiple diverse HA antigens on a self‐assembling nanoparticle [182, 183]. Notably, vaccinations with such mosaic nanoparticles elicit broader humoral responses than cocktails of nanoparticles each displaying a single HA antigen. The epitope valency and density on a mosaic nanoparticle are mechanistically higher for conserved epitopes across diverse antigens than for strain‐specific epitopes. This vaccine design embodies an immunologically intriguing model in which broadly reactive B cells acquire avidity through bivalently engaging conserved epitopes, thereby enabling preferential selection.

**FIGURE 6:**
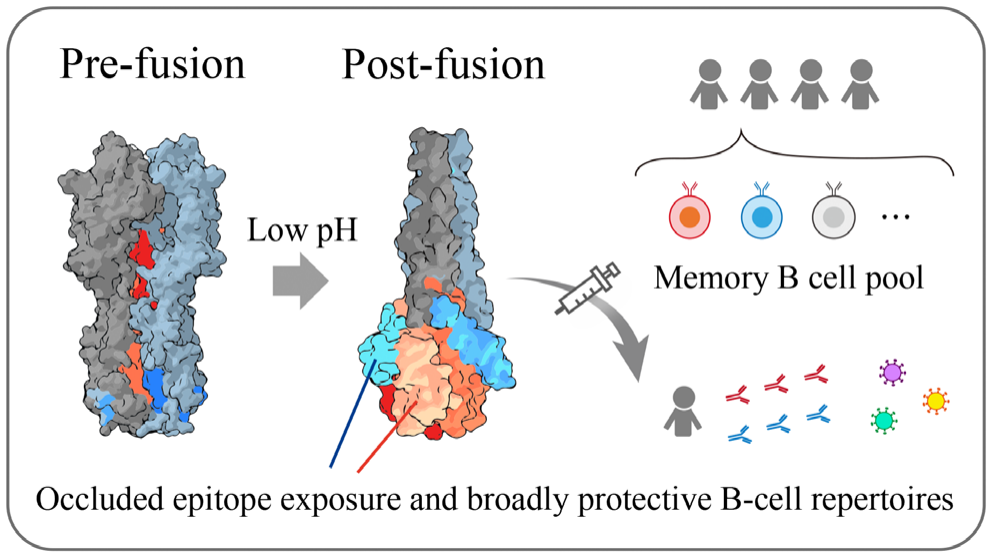
Post‐fusion HA vaccine. The epitopes on individual protomers are shown in distinct shades of red (LAH epitope) or blue (β‐hairpin epitope) on the pre‐fusion HA trimer (PDB 3VUN) and the post‐fusion HA trimer (PDB 1HTM).

Previous efforts have exclusively focused on vaccine designs that increase the immunodominance of conserved epitopes, such as the CS epitope displayed on the pre‐fusion HA surface or the head‐interface epitope partially occluded in the pre‐fusion HA. However, the discovery of multiple broadly protective epitopes that are absent or totally hidden under the native pre‐fusion structure has prompted the development of vaccine strategies that do not rely on the natural HA architecture. Among the conceptually shifted strategies of vaccine designs, a representative approach utilizes post‐fusion HA as vaccine immunogen (Figure 6). This vaccine antigen is produced by introducing an acid‐treatment step into the standard manufacturing process for split vaccines, requiring only modest modifications to conventional production methods.

Post‐fusion vaccines were originally conceived to induce LAH antibodies, and indeed, they have been shown to elicit both GC B cell responses and antibody production targeting LAH epitopes, leading to heterosubtypic protection in the mouse model [109, 184]. LAH antibody repertoire constitutes a relatively large proportion of broadly reactive antibodies within the human memory B‐cell pool. LAH antibody clones include LAH31, which shows inter‐group reactivity [52], as described in Chapter 4. Importantly, the immunogenic potential of post‐fusion HA may not be limited to the LAH epitope. This vaccine may elicit antibody responses to the β‐hairpin epitope. Indeed, immunization with EHA2 [185], which is a headless HA with a post‐fusion‐like structure, has been shown to induce the production of influenza B‐reactive antibodies in mouse models [186]. Antibody clones corresponding to this β‐hairpin antibody repertoire are also readily detectable within the human memory B‐cell compartment. Despite the relative abundance of such broadly protective antibody repertoires in human memory B cells, routine vaccination cannot elicit LAH antibodies [187]. Taken together, these findings suggest that the post‐fusion HA vaccine has the potential to activate and further mature pre‐existing broadly reactive antibody repertoires.

### 6.1 How Can We Estimate the Contribution of Non‐Neutralizing Antibodies to Protection?

Serum HAI titers remain the primary CoP to evaluate licensed influenza vaccines. Historically, the HAI titer of approximately 1:40 in serum has been associated with a 50% reduction in the risk of developing clinical influenza illnesses. Consequently, seroprotection and seroconversion rates based on HAI titers are widely adopted as regulatory criteria for vaccine approval [188]. However, the protective threshold defined by HAI titers varies considerably across age groups and epidemiological settings. Furthermore, HAI titers do not encompass the multiple protective mechanisms by non‐neutralizing antibodies described in Chapter 2. Therefore, recent findings underscore the limitations of HAI titers as a universal CoP and highlight the need for more comprehensive and relevant immunological metrics to evaluate next‐generation influenza vaccines.

Recent vaccine strategies aim to elicit broadly protective antibodies that often display limited or undetectable neutralizing activity yet confer protection against antigenically divergent influenza viruses. Therefore, correlates that rely exclusively on neutralization readouts, such as HAI activity, may substantially underestimate the protective potential of vaccine‐elicited antibodies. To address this gap, several Fc‐function–associated indices have been proposed as emerging CoP candidates for next‐generation vaccines. These include quantitative measurements of serum ADCC, ADCP, and CDC activities; frequencies and activation profiles of effector cell populations such as NK cells and CD4^+^/CD8^+^ T cells that reflect local immunity at the site of infection [189]. However, these assays are not yet standardized across laboratories, and substantial methodological variability prevents their use as regulatory endpoints. Assay harmonization and the definition of clinically meaningful thresholds will be essential for their future implementation.

Among the candidates for revised CoP, serum antibody titers specific to the target epitope may provide a more reliable predictor of the efficacy of next‐generation vaccines, aiming for intensive on‐target antibody responses. Clinical evidence supporting this concept is obtained in part from the A/H1N1 influenza pandemic in 2009 [162]. During this pandemic, older adults exhibited comparatively lower infection rates than younger individuals [190, 191, 192]. Retrospective seroepidemiological studies indicated that older adults possessed pre‐existing cross‐reactive antibodies recognizing conserved HA stalk regions/epitopes, despite low HAI titers to the H1N1pdm09 virus [193, 194, 195]. Those findings highlight the potential utility of epitope‐specific antibody titers to complement the CoP using conventional HAI titers.

## 7 Perspective

The decades‐long work has culminated in massive advances in technology that have deepened and broadened our understanding of immunological insights, driving progress in next‐generation vaccine development. Recent remarkable advances in IT technologies, such as artificial intelligence (AI), machine learning, and immunoinformatics, are expected to further accelerate rational vaccine design in the near future [196]. High‐throughput deep mutational scanning (DMS) can comprehensively predict viral escapes under immune pressure from existing protective antibodies through prior infection or vaccination, providing key insights into viral antigens for vaccine design [197]. Additionally, the development of humanized mouse technologies, such as the human BCR knock‐in mouse [198] and the human V(D)J‐rearranging mouse [199], now enables the modeling and evaluation of human immune responses to vaccination, which could not be assessed using conventional experimental animals.

Furthermore, the global COVID‐19 pandemic, caused by the emerging severe acute respiratory syndrome coronavirus 2 (SARS‐CoV‐2) in 2019 [200], led to the authorization of several novel vaccine platforms for human use. mRNA vaccines were rapidly authorized upon the COVID‐19 outbreak, and clinical trials are now underway to evaluate their use in influenza vaccines as well [201, 202]. Furthermore, self‐amplifying RNA (saRNA, also known as a replicon) vaccines have been approved in Japan and India [203, 204]. In clinical studies, saRNA vaccine candidates have demonstrated higher seroresponse rates and more durable antibody titers than approved mRNA or adenovirus vector vaccines [205, 206, 207], presumably due to the sustained antigen supply from saRNA, which may support persistent GC responses. The application of the saRNA vaccine platform to influenza is currently being evaluated in animal models, including mouse [208], bird [209], and pig [210].

A key outstanding challenge that remains is how to confer durable and broadly protective immunity against antigenically divergent viruses across diverse human populations. In addition to the B cell and antibody profiling, we also need a deeper understanding of T cell repertoires and human leukocyte antigen (HLA) diversity, both of which are essential for shaping protective antibody responses to vaccination [211]. Therefore, by fully leveraging broad knowledge on human immunity through state‐of‐the‐art technologies, we can develop a more comprehensive approach to counter influenza viruses through the full spectrum of protective antibodies.

## Funding

This work was supported by JSPS KAKENHI Grant Number 25K22567, Japan Agency for Medical Research and Development JP25gm1810004, JP253fa627005, JP253fa627009, and JP253fa727002.

## Conflicts of Interest

Y.A. and Y.T. are inventors on patents related to the work described in this manuscript.

## Data Availability

Data sharing is not applicable, as no new data are included in this review.
